# Are Survivors Different? Genetic-Based Selection of Trees by Mountain Pine Beetle During a Climate Change-Driven Outbreak in a High-Elevation Pine Forest

**DOI:** 10.3389/fpls.2018.00993

**Published:** 2018-07-23

**Authors:** Diana L. Six, Clare Vergobbi, Mitchell Cutter

**Affiliations:** ^1^Department of Ecosystem and Conservation Sciences, University of Montana, Missoula, MT, United States; ^2^Department of Biology, Whitman College, Walla Walla, WA, United States

**Keywords:** *Pinus albicaulis*, *Pinus contorta*, *Dendroctonus ponderosae*, whitebark pine, climate adaptation, climate change, natural selection

## Abstract

Increased mortality of forest trees, driven directly or indirectly by climate change, is occurring around the world. In western North America, whitebark pine, a high elevation keystone species, and lodgepole pine, a widespread ecologically and economically important tree, have experienced extensive mortality in recent climate-driven outbreaks of the mountain pine beetle. However, even in stands experiencing high levels of mortality, some mature trees have survived. We hypothesized that the outbreak acted as a natural selection event, removing trees most susceptible to the beetle and least adapted to warmer drier conditions. If this was the case, genetic change would be expected at loci underlying beetle resistance. Given we did not know the basis for resistance, we used inter-simple sequence repeats to compare the genetic profiles of two sets of trees, survivors (mature, living trees) and general population (trees just under the diameter preferred by the beetles and expected to approximate the genetic structure of each tree species at the site without beetle selection). This method detects high levels of polymorphism and has often been able to detect patterns associated with phenotypic traits. For both whitebark and lodgepole pine, survivors and general population trees mostly segregated independently indicating a genetic basis for survivorship. Exceptions were a few general population trees that segregated with survivors in proportions roughly reflecting the proportion of survivors versus beetle-killed trees. Our results indicate that during outbreaks, beetle choice may result in strong selection for trees with greater resistance to attack. Our findings suggest that survivorship is genetically based and, thus, heritable. Therefore, retaining survivors after outbreaks to act as primary seed sources could act to promote adaptation. Further research will be needed to characterize the actual mechanism(s) of resistance.

## Introduction

The capacity of forests to adapt to rapid climate change is not known. Their ability to adapt will vary greatly depending upon tree species, amount and type of genetic variation existing within and among populations, type and degree of change required, strength and type of selection pressure, heritability of desirable traits, and the timeframe over which selection is able to act. Many long-lived sessile organisms, including trees, are unlikely to be able to track shifting conditions through migration (Kremer et al., 2012). This is especially true for those restricted to montane ecosystems where movement higher in elevation ends at the top of the mountain and poleward migration is blocked by competitors, valleys, and development (Jump and Penuelas, 2005; Aitken et al., 2008; Dullinger et al., 2012). For many tree species and forests, adaptation will need to occur in place if they are to persist into the future (Aitken et al., 2008).

Bioclimatic envelope models used to predict range expansions and contractions of forest trees treat species as clones, with all individuals exhibiting identical responses (Mimura and Aitken, 2007). While these models are useful to provide estimates of shifts in habitat suitability, they can mask the high genetic diversity and geographic differentiation of most tree species (Mimura and Aitken, 2007; Thuiller et al., 2008; Reed et al., 2011). Likewise, most management focuses primarily on increasing forest resilience through manipulating stand structure and composition while ignoring genetic diversity, natural selection, and the potential for adaptation (Churchill et al., 2013; O’Hara and Ramage, 2013; DeRose and Long, 2014).

Except for highly fragmented or relictual populations, forest trees possess moderate to high levels of standing genetic variation and often exhibit considerable local adaptation and within and among population diversity (Austerlitz et al., 2000; Hamrick, 2004; Savolainen et al., 2007; Alberto et al., 2013). Adaptation of forests to climate change will depend upon the outcome of interactions between existing genetic diversity, phenotypic plasticity, and selection pressure over a relatively short period of time. However, adaptation in trees can be slow due to long generation times and low mortality of older, well-established, but increasingly maladapted trees that continue to contribute to the gene pool (Savolainen et al., 2007; Kuparinen et al., 2010). Long generation times can result in considerable genetic load with long lags between mean optimal genotype and existing climate (Kuparinen et al., 2010). Additionally, while phenotypic plasticity may allow some genotypes to maintain high fitness over a broad range of environmental conditions and aid in resilience to climate change in the short-term, it may slow down or hinder adaptation and persistence in the longer-term (Valladeres et al., 2014).

Adaptation in trees may be accelerated when new conditions or agents lead to high levels of mortality and directional selection in favor of heritable traits associated higher fitness and survival. For example, Kuparinen et al. (2010) used computer simulations to investigate rates of adaptation to longer thermal growing seasons and found that mortality of established trees was the key factor regulating the speed of adaptation with dispersal ability and maturation age having substantially lesser effects. Disturbances caused by agents that use selective behaviors in choosing individual trees, such as herbivorous insects that respond positively to tree stress, can elicit rapid microevolution even in slow-growing tree species (Petit and Hampe, 2006). Such agents may benefit forests in the long-term by increasing mortality of poorly adapted trees, enhancing the reproductive potential of surviving better-adapted trees, and reducing genetic lag loads in affected populations (Kuparinen et al., 2010; Pedlar and McKenney, 2017).

The mountain pine beetle (MPB) (*Dendroctonus ponderosae*) is a highly selective insect that chooses hosts based on a complex array of chemical cues whose production by the tree is influenced by both tree condition and genotype (Emerick et al., 2008; Blomquist et al., 2010). Secondary metabolic chemicals produced by the tree are used by MPBs to distinguish among tree species as well as to assess the relative strength of defenses of individuals (Blomquist et al., 2010; Raffa et al., 2017). Such chemicals also likely signal adequacy of nutritional content for brood production given that beetles avoid hosts of very poor quality (Taylor et al., 2006; Dooley et al., 2015). Using such cues, a MPB will decide whether or not to enter a particular tree and initiate a mass attack. Once in the tree, the insect converts some terpenes to pheromones important in initiating and sustaining the mass attack required to kill the tree (Blomquist et al., 2010). When MPB populations are low to moderate in size, weakened trees with poor defenses that require fewer beetles to overcome defenses are most often attacked (Boone et al., 2011). However, during outbreaks, MPBs may switch to attacking healthier trees that, although better defended, possess thicker phloem and higher nutritional contents for brood development (Boone et al., 2011). Interestingly, some trees escape attack even when MPB populations are present in high numbers and suitable hosts become increasingly scarce.

In this study, we investigated whether trees that survive MPB outbreaks are genetically different than those that are selected for colonization and killed. Our overarching hypothesis was that surviving trees do not escape by chance, but rather possess genetically based characteristics that confer resistance. The basis for resistance, whether it is the ability to tolerate warmer drier conditions without a reduction in defenses, a chemical profile that negatively affects MPB host location or selection, or some other phenotypic trait, is likely to be under genetic control (González-Martínez et al., 2006; Keeling and Bohlmann, 2006).

MPB outbreaks are triggered by extended periods of warm weather and drought (Meddens et al., 2012). The recent MPB outbreak in western North America was a magnitude larger than any recorded in the past and affected millions of hectares of pine forest (Meddens et al., 2012). The outbreak was primarily driven by climate although its severity was intensified in some areas by past logging practices and fire suppression (Taylor et al., 2006; Creeden et al., 2014; Buotte et al., 2017). Climate change also supported movement of MPB further north in British Columbia and eastward across Alberta into naïve forests (those with no prior history of MPB) of lodgepole pine and jack pine (*P. banksiana*, a novel species for MPB) (Burke et al., 2017). While the size and extent of the recent outbreak was far outside the historic norm, outbreaks of MPB are not unusual and have likely occurred for millennia. Selection by MPB during outbreaks, as well as persistent low-level activity during non-outbreak periods, are believed to have been a major force shaping constitutive and induced defenses in host pines (Raffa and Berryman, 1987; Franceschi et al., 2005). MPB activity in naïve forests can be expected to exert especially rapid and strong selection for host resistance because of high levels of susceptibility. Indeed, naïve lodgepole and jack pine forests exhibit lower defenses to MPB attack than those with a co-evolutionary history with the beetle (Clark et al., 2010; Cudmore et al., 2010; Raffa et al., 2013, 2017; Burke et al., 2017).

We focused on two tree species that have suffered high mortality by MPB in the recent outbreak. One is a relatively naïve host, whitebark pine (*Pinus albicaulis*), and the other is a highly co-evolved host, lodgepole pine (*P. contorta*). Whitebark pine is a high elevation tree that is considered a keystone in western subalpine ecosystems of the United States and Canada (Tomback et al., 2016). Historically, outbreaks in whitebark pine appear to have been rare and limited in size (Logan et al., 2010). During warm periods, beetles sometimes moved upslope from lower elevation outbreaks (Bartos and Gibson, 1990) where they killed some whitebark pine, but either did not reproduce successfully due to winter mortality, or completed only one or a few generations before the return of cold conditions once again limited them to lower elevations (Logan et al., 2010). The recent outbreak in whitebark pine has been extensive and has been driven by chronic warm temperatures that allowed the beetle to move into the subalpine and to persist there for an extended period (Buotte et al., 2016, 2017). With climate change, the presence of MPB in high elevation whitebark pine forests is expected to be persistent rather than occasional (Buotte et al., 2016, 2017). Whitebark pine exhibits many of the characteristics of a naïve host, including lower levels of defense chemicals and resin (Raffa et al., 2013, 2017). Reduced snow packs may also result in greater drought stress that may increase susceptibility (Larson and Kipfmueller, 2012). Outbreaks in this tree have been devastating in some areas, including the Greater Yellowstone Area, contributing to the recommendation that it be listed as an endangered species (United States Fish, and Wildlife Service [USFWS], 2011).

The second species studied was lodgepole pine, a co-evolved host that has experienced repeated extensive outbreaks in much of its range, likely over a long evolutionary period. Vigorous lodgepole pine typically exhibits strong constitutive and inducible defensive responses to beetle attack (Burke et al., 2017; Raffa et al., 2017). Outbreaks of MPB in lodgepole pine are considered natural disturbances that, much like fire, help maintain lodgepole pine forests by periodically regenerating new stands free of many diseases, initiating nutrient cycling, and stimulating regeneration, understory productivity, and supporting biodiversity (Dordel et al., 2008; Diskin et al., 2011; Pec et al., 2015).

Our objective in this study was to investigate whether whitebark and lodgepole pine growing in a mixed high elevation stand that survived the outbreak are genetically distinct. If so, this may indicate an increased potential for these pines to persist in the face of the more frequent and extensive outbreaks predicted due to a changing climate. We would expect genetic change at loci underlying beetle resistance but not at a genome-wide scale. Without knowing the basis for resistance in survivors, we chose to use inter-simple sequence repeats (ISSRs) to develop genetic profiles for whitebark and lodgepole pine. ISSRs target highly variable sequences within microsatellite regions (Parasharami and Thengane, 2012). Because ISSR markers can be used to detect high levels of polymorphism and are highly reproducible, they provide a powerful approach for comparing genetic diversity between individuals as well as within and among populations of plants including pines (Mehes et al., 2007; Parasharami and Thengane, 2012; Lucas-Borja et al., 2016). In many studies, ISSR profiles have been useful in marker assisted selection when particular markers were associated with particular traits (REFS). In our screens, we looked for patterns that indicted differences between survivors and susceptible trees.

## Materials and Methods

### Site Description

This study was conducted at Vipond Park, a high-elevation plateau supporting a patchwork of grassland and open forest stands located on the Beaverhead National Forest, Montana, United States (2,501 m elevation, 45.6974^o^N, 112.9106^o^W). The site is relatively xeric with an understory of sagebrush and a diverse mixture of annual and perennial forbs. Vipond Park was chosen to take advantage of the high mortality to pines that occurred there during a recent high elevation outbreak of MPB (2009–2013) when approximately 93 and 75% of mature *P. albicaulis* and *P. contorta*, respectively, were killed. The relatively flat topography of the plateau combined with its location at the transition zone between lodgepole and whitebark pine-dominated forests allowed us to study the effects of MPB selection on more than one pine species growing under the same conditions and experiencing the same level of beetle pressure. Although *P. contorta* existed at lower numbers than *P. albicaulis* at the site, they were abundant enough to allow sufficient sampling to make comparisons with whitebark pine. Additionally, white pine blister rust infection incidence and severity were very low reducing the potential for the presence of the disease to influence the choice of individual host trees by the beetle (Six and Adams, 2007).

### Transects

Transects were established in 2015 (*P. albicaulis*) and 2016 (*P. contorta*). These were variable length belt transects 2 m in width that started on the edge of a stand and then followed a randomly chosen bearing until another edge was reached at which point a new bearing was adopted to establish a new transect in the same or an adjacent stand. This process was continued until the desired number of trees per species per treatment were measured. When trees occurred in clumps (resulting from seed caching by Clark’s Nutcrackers), we restricted measurements and samples to one tree per clump to avoiding sampling trees potentially originating from the same cone/parent.

### Determination of the Diameter Distribution of Mountain Pine Beetle-Killed Pines

In initial transects, the diameter at breast height (DBH, 1.4 m above the soil line) of 100 *P. albicaulis* and 45 *P. contorta* killed by MPB were measured to estimate the diameter distribution of MPB-killed trees for each species. This distribution was used to inform our sampling of “survivors” (mature trees that survived the outbreak) so that a similar distribution was achieved, and to determine the diameter below which trees were not attacked.

### Collection of Samples for Genetic Analysis

In 2015, transects were established as previously described. Thirty survivor *P. albicaulis* with diameters representative of the diameter distribution of MPB-killed *P. albicaulis* were located on the transects. For each tree, DBH was measured and each was rated for white pine blister rust infection severity using the method of Six and Newcomb (2005). Then, approximately 30 current-year needles were collected and placed in a small plastic bag that was sealed and placed on ice in a cooler. In the lab, needles were placed into silica gel for drying and preservation. In 2016, this procedure was repeated for *P. contorta* (*n* = 20) (except for rust rating) in the same stands sampled the previous year.

The smallest diameters of *P. albicaulis* and *P. contorta* killed by MPB were 12 and 18 cm, respectively. Because beetle-killed trees did not yield DNA, we used this information to choose a second set of living trees for sampling of each species we designated as the “general population.” These trees were expected to approximate the genetic structure of the population of each tree species at the site without beetle selection and so should contain a mix of survivor and “susceptible” genotypes. If our hypothesis was correct that survivors were genetically distinct from beetle-susceptible trees, then we expected only a few general population trees would have genotypes matching those of survivors (roughly reflecting the proportion of mature survivors to mature MPB-killed trees at the site). To sample general population trees, we established similar transects as before, but collected needles from trees between 9–11 and 14–17 cm DBH for *P. albicaulis* (*n* = 36) and *P. contorta* (*n* = 20), respectively.

### DNA Extraction and Amplification

Needles (3–5) from each sample were ground to a fine powder in liquid nitrogen using a mortar and pestle. DNA was then isolated from each sample using a Qiagen DNeasy Plant Kit (Qiagen, Valencia, CA, United States) following the protocol provided by the manufacturer.

Five ISSR primers were chosen for use (**Table 1**). Not all primers worked equally well for both species of trees. Therefore, we chose three primers for use with *P. albicaulis* and four for *P. contorta*. Two primers overlapped in use for both trees (**Table 1**).

**Table 1:** Primers used for ISSR amplification.

Primer ID	Sequence	Tree species
HB12	CAC CAC CAC GC	*Pinus albicaulis*
17899A	GTG TGT GTG TGT CA	*P. albicaulis, P. contorta*
17901	CAC ACA CAC ACA AG	*P. contorta*
UBC 807	AGA GAG AGA GAG AGA GT	*P. albicaulis, P. contorta*
UBC 811	GAG AGA GAG AGA GAG AC	*P. contorta*


For amplification we used a 25 μl reaction mixture consisting of 12.5 μl Promega Master Mix (Promega, Madison, WI, United States), 2.5 μl RNA-free water, 8 μl of 0.5 M primer and 2 μl of DNA template. Reactions were run individually with one of the five ISSR primers. PCR was conducted with one cycle denaturation at 95°C for 5 min, followed by 42 cycles of denaturation at 95°C for 1.3 min, annealing at 47°C for 2 min, and extension at 72°C for 1 min. A final cycle was conducted at 72°C for 1 min and final products were held at 6°C (Parasharami and Thengane, 2012).

PCR products were visualized in a 1% agarose gel prepared using 1× tris borate buffer (TBE) to which 2 μl ethidium bromide per 100 ml gel was added. A 100 bp ladder (Promega, Valencia, CA, United States) was placed in the first lane of each gel to provide a reference for scoring bands. Amplified DNA was loaded into the remaining lanes with bromophenol blue as a running dye. Each gel was run with 1× TBE as a running buffer at 70 mA until the dye moved 3/4 of the length of the gel. Gel images were captured using a UV table. Any sample that gave ambiguous results (no, faint, or smeared bands) was repeated. Approximately 20% of samples were rerun and compared to check for consistency in results. Only samples exhibiting clear bands were included in the final analysis. Bands were scored manually.

### Data Analysis

#### Diameter Distributions

A two-sample *t*-test was used to compare mean diameters among groups (survivor, general population, and beetle-killed) using Statistix 7 (Analytical Software, Tallahassee, FL, United States).

#### Genetic Analysis

Bands were scored as present (1) or absent (0) to develop a binary matrix combining data for all primers by tree species. The matrices were analyzed in Popgene v. 1.32 (Yeh et al., 1997) (assuming each group was in Hardy-Weinberg equilibrium) to calculate percent polymorphism, the Shannon information index (*I*), Nei’s gene diversity index (*h*), total genetic diversity (*H*_T_), genetic diversity within groups (survivor, general population) (*H*_S_), and evidence for deviations from neutrality (selection) with an overall Ewens–Watterson test for neutrality. Population genetic structure was investigated using STRUCTURE v. 2.3 (Pritchard et al., 2000). The *admixture* model was used with a 10,000 burn-in period and 10,000 Markov Chain Monte Carlo replications. Twenty runs were performed with each value from 1 to 10 to estimate the optimal number of clusters (K) using the ΔK statistic (Evanno et al., 2005).

For each tree species, we examined genetic variation between groups using analysis of molecular variation (AMOVA) in GenAlEx 6.5 (Peakall and Smouse, 2006). We then conducted a principle coordinates analysis (PCoA) in GenAlEx based on genetic distances between individual trees in the two groups for each species of tree. Genetic distance matrices were developed for each tree species in the *Restml* program and then imported into *Neighbor* in PHYLIP 3.67 (Felsenstein, 2005) to produce an unweighted neighbor-joining tree. The tree was visualized using TreeView 1.6.6 (Page, 1996).

## Results

### Diameter Distributions and Blister Rust Infection Severity

The mean, median, and range of diameters of beetle-killed and survivor *P. albicaulis* were similar (**Table 2**). The mean diameter was not significantly different between survivor and beetle-killed trees, while the diameter of general population trees, as expected, differed significantly from both groups (**Table 2**). The same was true for *P. contorta* (**Table 2**). Similarly, mean diameters of MPB-killed and survivor *P. albicaulis* and *P. contorta* did not differ from one another. However, the minimum size of tree attacked by the beetle differed by tree species resulting in the choice of different diameter distributions for sampling general population trees (**Table 2**). Blister rust infection severity was overall very low at the site, but significantly lower in survivors (mean = 1.3, *SD* = 1.8) than in general population trees (mean = 1.7, *SD* = 2.4; *F* = 1.63, *df* = 65, *P* = 0.013; potential range 0–18).

**Table 2:** Summary statistics for diameter breast height (cm) of *Pinus albicaulis* and *P. contorta* by group.

Tree	Group	N	Mean (*SD*)	Median	Minimum	Maximum
*P. albicaulis*	Beetle-killed	75	24.5 (5.3)^a^	24.2	12.0	37.3
	Survivor	30	25.0 (5.2)^a^	24.1	17.0	37.3
	General	36	10.0 (0.6)^b^	10.0	9.0	11.0
*P. contorta*	Beetle-killed	45	26.7 (5.0)^a^	26.4	17.5	36.8
	Survivor	20	27.5 (5.4)^a^	29.9	18	37.2
	General	20	15.3 (0.9)^c^	15.2	13.9	16.8


*Different letters following means denote statistically significant differences, α = 0.05.*

### Genetic Analyses

#### *Pinus albicauli*s

Three primers (17899A, HB12, and UBC807) resolved well for *P. albicaulis* and were used for ISSR analysis. A total of 28 loci (bands) were resolved using the three primers (**Table 3**). Mean percent band polymorphism (BP) for all primers for all trees (general population and survivors) combined was 96.4% and this value was similar to the BP for each group individually. The Shannon information index and Nei’s gene diversity was lower in general population trees compared with survivors (**Table 2**). Nei’s unbiased measure of genetic identity between the survivor and general population trees was 95% while genetic distance was a corresponding 5%.

**Table 3:** Percent band polymorphism (BP), number of observed (N_a_) and effective (N_e_) alleles, Shannon’s Information Index (*I*), Nei’s gene diversity (*h*), and diversity between (H_T_) and within groups (H_S_), presented by tree species and group.

Tree species	Group	N	%BP	N_a_	N_e_	*I*	*h*	H_T_	H_S_
*P. albicaulis*	Survivor	30	96.58	1.97 (0.19)	1.39 (0.25)	0.40 (0.19	0.22 (0.14)		
	General	36	96.55	1.97 (0.19)	1.32 (0.27)	0.36 (0.14)	0.25 (0.14)		
	Combined	66	96.43	1.96 (0.19)	1.41 (0.19)	0.42 (0.18)	0.26 (0.14)	0.26 (0.10)	0.24 (0.01)
*P. contorta*	Survivor	20	88.24	1.88 (0.32)	1.40 (0.33)	0.25 (0.17)	0.39 (0.23)		
	General	20	89.41	1.89 (0.31)	1.40 (0.31)	0.26 (0.16)	0.40 (0.22)		
	Combined	40	98.82	1.90 (0.11)	1.41 (0.29)	0.27 (0.14)	0.42 (0.18)	0.27 (0.02)	0.25 (0.02)


*Means are accompanied by standard deviations in parentheses.*

H_T_, the total genetic diversity between the two study groups, was 0.26, and the diversity within groups, H_S_, was 0.24. Seven of 28 loci (25%) exhibited significant differences between observed and expected frequencies of bands between the two groups (data not shown). However, no bands were unique to either group. The Ewens–Watterson test for neutrality detected only one marginally non-neutral locus. AMOVA indicated 87% of the variation exhibited existed within groups and 13% existed between groups.

**FIGURE 1:**
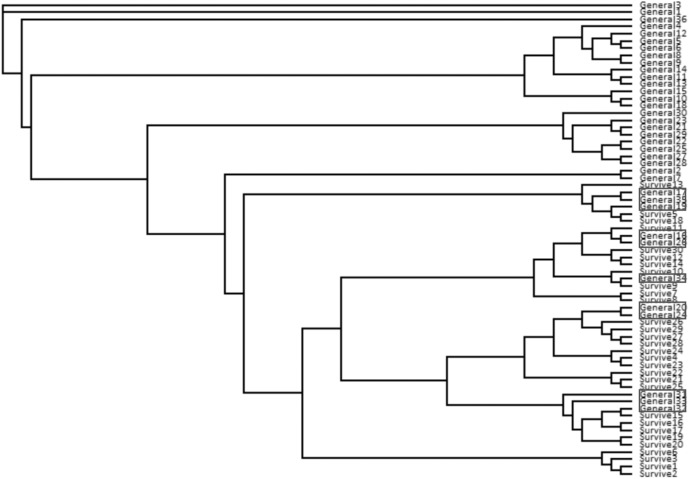
Neighbor-joining tree from ISSR data for *Pinus albicaulis*. General = general population trees (with no *Dendroctonus ponderosae* selection). Survive = mature trees surviving *D. ponderosae* outbreak. Trees in boxes correspond to trees with arrows in **Figure 2** and in ellipses in **Figure 3**.

**FIGURE 2:**
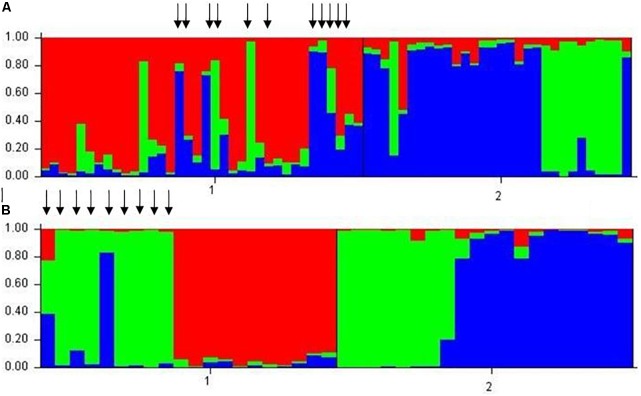
Results of Bayesian clustering using STRUCTURE. Individual trees are represented by vertical bars. Colored segments represent the tree’s estimated proportion similarity to each of the three clusters (red, blue, and green) optimally defined by STRUCTURE. **(A)** *Pinus albicaulis*. Arrows denote general population trees that resolved with survivors in neighbor-joining tree in **Figure 1**. **(B)**. *Pinus contorta*. 1 = general population trees. 2 = survivor trees.

**FIGURE 3:**
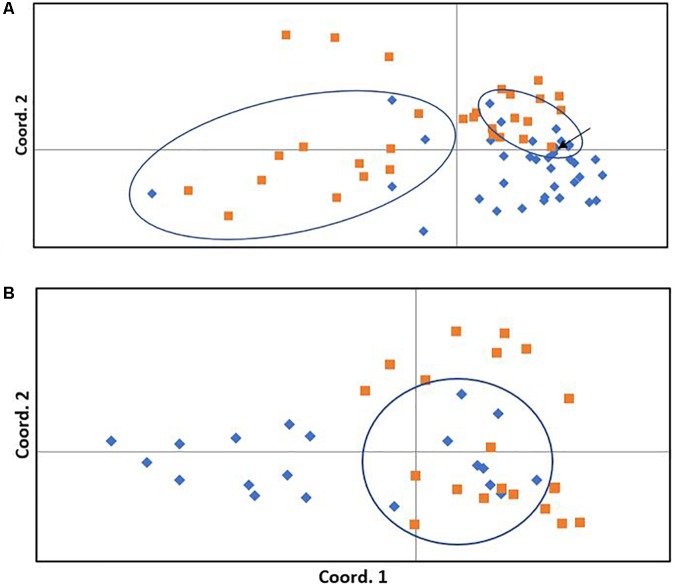
Principle coordinates analysis of general (blue diamonds) and survivor (orange squares) trees. **(A)** *Pinus albicaulis*. The first and second coordinates explain 19.29 and 13.67% of the variation among trees, respectively (total 33%). **(B)** *Pinus contorta*. The first and second coordinates explain 10.98 and 10.55% of the variation among trees, respectively (total 21.5%). Ellipses surround general population trees that clustered with survivors in the neighbor-joining tree (**Figure 1** for *P. albicaulis*, **Figure 4** for *P. contorta*) and correspond to trees marked with an arrow in the STRUCTURE analysis (in this figure). Arrow indicates one general population tree within the ellipse that did not cluster with survivors in the neighbor-joining tree.

The neighbor-joining tree resolved most general population trees together in the basal clades while one major terminal clade contained all survivor trees as well as eleven general population trees that were distributed throughout the clade (**Figure 1**). The results of Bayesian clustering using STRUCTURE indicated that the optimal *K*-value was 3 with the general population dominated by one cluster (red, **Figure 2**) and survivors dominated by the other two (blue and green, **Figure 2**). The eleven general population trees that clustered with survivor trees in the neighbor-joining tree exhibited predominantly blue and green profiles in the STRUCTURE bar graph (shown with asterisks) indicating similarity to survivors (**Figure 2**). In the PCoA, the first two principle coordinates explained a total of 33% of the variation associated with the two groups. Adding the third, 43.55% was explained. In general, the eleven general population trees that clustered with survivors in the neighbor-joining tree resolved separate from other general population trees and with survivors in the PCoA (**Figure 3**).

#### Pinus contorta

Four primers resolved well for this species (17899A, UBC807, UBC901, and UBC811). Using these primers, we were able to resolve a total of 85 bands. The mean percent BP across all primers and groups was 98.82. This was considerably higher than BP for the general population (89.4%) and survivor (88.2%) trees (**Table 2**). The mean number of effective alleles was slightly lower than the mean number of observed alleles. Shannon’s information index was similar within and across groups while Nei’s gene diversity was lowest in survivors and highest for both groups combined (**Table 2**). Nei’s unbiased genetic identity and diversity between the two groups was 93 and 7%, respectively.

H_T_ was 0.26 and H_S_ was 0.25, similar to values for whitebark pine. Allele frequencies were significantly different between survivors and general population trees at 12 of 85 loci (14%) (**Table 3**). No bands were unique to either group. The Ewan–Watterson test for neutrality indicated that six loci in the general population and nine loci in the survivors were outside the 95% CI indicating non-neutrality. All had positive *F*-values greater than the upper bound indicating a potential for directional selection. AMOVA indicated that 89% of variation occurred within groups while 11% occurred between groups.

**FIGURE 4:**
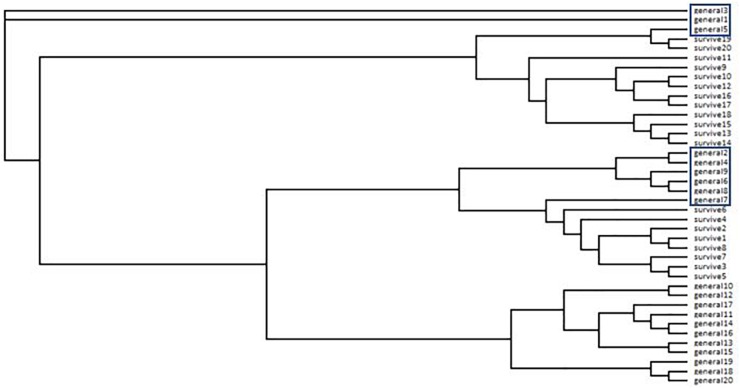
Neighbor-joining tree from ISSR data for *Pinus contorta*. General = general population trees (with no *Dendroctonus ponderosae* selection). Survive = mature trees surviving *D. ponderosae* outbreak. Trees in boxes correspond to trees with arrows in **Figure 2** and in ellipses in **Figure 3**.

The neighbor-joining tree partitioned general population and survivor trees into several clades (**Figure 4**). Most (55%) general population trees resolved in one clade. The remainder resolved into two clades interspersed with survivors (**Figure 4**). The general population trees that resolved with survivors in the neighbor-joining tree shared clusters with survivor trees in the STRUCTURE analysis (**Figure 2**) and also partitioned with survivor trees in the PCoA (**Figure 3**). The first two principle coordinates in the PCoA explained 21.5% of the variation between the two groups. Adding the third component explained 31%.

## Discussion

Knowledge of the effects of bark beetle outbreaks on host tree population genetic structure and resistance to attack will be increasingly valuable as climate change drives more frequent outbreaks and facilitates the movement of beetle species into naïve forests. Outbreaks of MPB seldom kill all mature trees despite high beetle numbers during population peaks. Our results suggest that surviving trees possess a wealth of information that can be used to inform our understanding of the genetic and phenotypic bases for resistance and to develop management approaches that support forest adaptation.

We found that surviving mature trees in a high elevation forest of whitebark and lodgepole pine were genetically distinct from “general population” trees that were assumed to represent the genetic structure of the population pre-outbreak and without selection by the beetle. In line with our hypothesis, a low percentage (<10%) of “survivor” genotypes were identified within the general population. The proportion of these survivors roughly mirrored the proportion of mature trees that survived the outbreak at Vipond Park. The neighbor-joining tree, the PCoA and the STRUCTURE analyses each indicated strong differentiation between survivors and “susceptible” individuals and identified the same trees as survivors within the general population. In the STRUCTURE analysis for both whitebark and lodgepole pine, susceptible trees belonged to one cluster while survivor trees belonged to two other clusters. This separation can also be seen in the PCoA. Further research will be needed to determine whether the patterns we detected are indeed indicative of resistance, and if so, whether there are multiple or overlapping factors that account for survivorship.

We found surprisingly high levels of differentiation between survivor and general population trees in both species of pine. For whitebark pine, Nei’s genetic distance between survivor and general population whitebark pines was 5%, a value that would indicate moderate differentiation if these comparisons had been made between tree populations. Likewise, AMOVA indicated 13% of the genetic variation present existed between groups. Considering that the trees in this analysis were not from different populations, but rather grew intermixed at the same site, these values seem strikingly high. Likewise, for lodgepole pine, Nei’s genetic distance was 7%, and AMOVA indicated 11% of variation occurred between the groups.

These results indicate the presence of genetically based resistance in both pine species and that trees with resistant genotypes are not selected for attack. It has been thought that once MPB achieve high population levels during outbreaks, the selection of individual trees based on tree-produced compounds and condition becomes swamped by high levels of aggregation pheromone production and competition for increasingly rare hosts (Safranyik and Carroll, 2006). However, our results suggest that beetles remain selective even as outbreaks peak and collapse.

We chose ISSR profiling as a first step to determine whether survivors were different than trees chosen by MPB for colonization. This PCR-based method detects high levels of polymorphism, is highly reproducible, and allows the screening of a large number of trees relatively rapidly and economically. Unfortunately, this method cannot tell us why survivors are different, only that they are. Further study will be needed to further investigate whether survivors are indeed highly resistant and, if so, to determine the actual basis behind resistance. Ongoing studies are investigating correlations among genetic profiles of survivor and “susceptible” trees with phenotypic traits including defensive chemistry and growth rates in relation to climate. Genomic approaches will also be extremely useful to elucidate the basis of resistance.

This study corroborated the findings of other studies that found that MPB colonizes smaller diameter whitebark pine than lodgepole pine during outbreaks (Dooley et al., 2015). The mortality of younger whitebark pine trees indicates a more severe impact of MPB outbreaks on whitebark pine forests, at least in the short term, because advanced regeneration is killed along with large trees. However, the loss of large and mid-diameter trees may serve to open areas for nutcracker caching of seeds from the remaining resistant trees, potentially increasing the frequency of those genotypes and phenotypes at the site and within the larger population.

In a previous study, Six and Adams (2007) found that as infection severity increased so did the likelihood of attack by the beetle. However, while we found that white pine blister rust infection severity was significantly higher in general population trees than survivors, the mean level of infection severity at the site was very low and the size effect between means for survivors and general population trees was very small. Therefore, we feel it is unlikely blister rust played a significant role influencing beetle dynamics at the study site.

A caution is in order in interpreting our results. We were unable to amplify DNA from MPB-killed trees which forced us to use smaller diameter “general population” trees as a substitution for “susceptible” trees. These trees were mature reproductive trees and only slightly smaller than trees selected by the beetle for colonization; however, some or all may constitute a cohort that regenerated under different environmental conditions resulting in a genetic structure unrepresentative of the larger trees that were available for selection by the beetle. However, the proportional distribution of survivor and “susceptible” trees in the neighbor-joining trees, PCoAs and STRUCTURE analyses indicate that the general population samples were likely appropriate proxies.

With climate change supporting the invasion of aggressive bark beetles into naïve forests, and predictions of more frequent and severe outbreaks, it is increasingly important to understand the capacity of trees to adapt and persist (Millar et al., 2007; Ramsfield et al., 2016). While the massive mortality of pines in western North America in recent years is cause for concern, we should also look at these hard-hit forests as opportunities to learn. In almost all cases, affected forests are not completely dead–they retain many living large diameter trees. If these trees are genetically different than those selected and killed by the beetles as our study suggests, these trees may aid in *in situ* adaptation and persistence. They may also be key to developing management and trajectories that allow for forest adaptation. For example, retaining surviving trees as a primary seed source, rather than removing them during salvage operations could support *in situ* adaptation. In contrast, the effects of natural selection in these stands could be instantly negated by clearcutting or replanting with general seed stock.

Supporting forest adaptation is critical in this time of rapid change (Millar et al., 2007). Given the great expanses of forest that are being affected by climate change and the fact that most will need to adapt *in situ*, it is imperative we begin to move past structural approaches to consider the genetic capacity of forest trees to adapt. The high degree of standing genetic variation found in most forest trees indicates many will have considerable ability to adapt. We need to be cognizant of adaptation that is occurring so that our management approaches act to support rather than hinder natural selection for traits needed under future conditions.

## Author Contributions

DS conceived of the project, participated in field work, conducted data analysis, and wrote the manuscript. CV conducted lab work on whitebark pine and participated in field work and data analysis. MC conducted field and lab work on lodgepole pine and contributed to data analysis.

## Conflict of Interest Statement

The authors declare that the research was conducted in the absence of any commercial or financial relationships that could be construed as a potential conflict of interest.
